# Biologicals

**DOI:** 10.1155/2012/892873

**Published:** 2012-03-22

**Authors:** Jozélio Freire de Carvalho, Simone Appenzeller, Yehuda Shoenfeld

**Affiliations:** ^1^Rheumatology Division, Universitary Hospital, Federal University of Bahia, 41810-080 Salvador, BA, Brazil; ^2^Rheumatology Division, Clinic Hospital of School of Medicnie from University of Campinas, 13083-887 Campinas, SP, Brazil; ^3^Zabludowitcz Center for Autoimmune Diseases, Sheba Medical Center, Tel-Hashomer, Sackler Faculty of Medicine, Tel Aviv University, 52621 Tel Aviv, Israel

Biological agents are currently a very important option for refractory autoimmune diseases [1]. From about ten years to now, several drugs are available to the clinical practice, mainly, in the rheumatology one. The main biological agents are antitumor necrosis factor (anti-TNF) medications, however there are other important drugs such as rituximab, which blocks lymphocyte B CD20, tocilizumab that inhibits anti-interleukin-6 receptor [2, 3]. In this special issue on biologicals from *Autoimmune Diseases* journal, we have invited several papers that address this modern issue. 

 The paper entitled “*Biological therapy systemic lupus erythematosus*” of this issue addresses the rational for the use of biological agents in patients with systemic lupus erythematosus. Several aspects including anti-CD20 and anti-CD22 antibodies, B-cell tolerogens, costimulatory blockers, anti-TNF, anti-interferon, anti-interleukins 1, 10, and 18, and also complement inhibitors. The paper entitled “Use of biological agents in ocular manifestations of rheumatic disease” reviews the different studies published in the literature regarding biological agents use on ocular disorders. 

 The paper entitled “*Tocilizumab for the treatment of rheumatoid arthritis and other systemic autoimmune diseases: current perspective and future directions*” is not only an elegant review of the use of tocilizumab in rheumatoid arthritis, but also in other autoimmune diseases such as lupus, systemic sclerosis, polymyositis and large vessel vasculitis.

Complications related to biologicals are discussed in this paper entitled “*Risk of orthopedic site surgical infections in patients with rheumatoid arthritis treated with anti-tumor necrosis factor alfa therapy*.” The authors performed a literature review of infections associated to orthopedic surgery in rheumatoid arthritis patients treated with anti-TNF and found inconclusive data in this field.

 An experimental study was presented on the paper entitled “*TNF-alpha in the locomotor system beyond joints: high degree of involvement in myositis in a rabbit model*.” In this research paper, the authors have evaluated the role of TNF in an experimental model of myositis and found interesting results.

The paper entitled “*Immunosuppresive exosome: a new approaching for treating arthritis*” brings a new methodology for treating arthritis. The rational consists that some vesicles derived from immunosuppressive dendritc cell may have immune suppressive properties and the authors reviewed several studies developed by themselves and others that used these vesicles (exosomes) as in experimental animals as in humans with the objective of treatment of arthritis.

*Jozélio Freire de Carvalho* *Jozélio Freire de Carvalho* *Simone Appenzeller* *Simone Appenzeller* *Yehuda Shoenfeld* *Yehuda Shoenfeld*
